# Initial intravenous immunoglobulin doses should be based on adjusted body weight in obese patients with primary immunodeficiency disorders

**DOI:** 10.1186/s13223-017-0220-y

**Published:** 2017-12-06

**Authors:** Rohan Ameratunga

**Affiliations:** Department of Clinical Immunology, Auckland Hospital, Park Rd, Grafton 1010, Auckland, New Zealand

**Keywords:** CVID, Bariatric surgery, IVIG, SCIG

## Abstract

**Background:**

Immunoglobulin therapy plays a critical role in the treatment of immunodeficiency disorders as well as autoimmune and inflammatory conditions. In immunodeficient patients, there has been controversy whether initial loading doses of intravenous (IVIG) should be based on actual body weight or a calculated parameter such as adjusted body weight in obese patients.

**Case presentation:**

I describe a patient with Common Variable Immunodeficiency disorder (CVID) who underwent bariatric surgery for morbid obesity. Her weight decreased by 50% to below her calculated ideal body weight (IBW) while her immunoglobulin requirement fell by approximately 20%. Her steady state serum IgG increased from approximately 7 g/l to 11.7 g/l concomitant with weight loss.

**Conclusions:**

I present this observation as support for the recommendation that initial loading doses of SCIG/IVIG in immunodeficiency should be based on adjusted body weight (AjBW) and not actual body weight in obese patients. This has important fiscal implications for treating obese patients with immunodeficiency disorders.

## Background

Immunoglobulin replacement is the standard of care for most patients with symptomatic primary immunodeficiency disorders (PIDs). Immunoglobulin replacement can be undertaken by either subcutaneous (SCIG) or intravenous (IVIG) routes. Current data suggests progressive improvement in health with gradually increasing serum IgG levels [1]. Generally, most patients become less symptomatic when their steady state/trough  IgG levels are kept above 7–8 g/l [2]. Many patient factors influence the steady state/trough serum IgG levels following SCIG/IVIG. These include the genotype of the FcRn receptor, the presence of infective or inflammatory conditions such as bronchiectasis. In most cases the maintenance immunoglobulin dose is subsequently adjusted, based on the clinical response [3, 4]. This has been referred to as the biological trough level [5, 6].

In patients with primary immunodeficiency disorders (PIDs) with profoundly low IgG levels, an initial loading dose of IVIG (1 g/kg) is often administered, followed by monthly maintenance doses of 400–800 mg/kg. A loading dose allows a patient to rapidly attain steady state therapeutic IgG levels, which would otherwise take several months, if typical maintenance doses were administered. There has been debate about whether the initial loading dose of IVIG in morbidly obese PID patients should be based on actual body weight or adjusted body weight [7]. Most clinical trials of IVIG therapy have excluded these patients.

In the event a loading dose is given, most experts recommend this initial SCIG/IVIG dose should be based on adjusted body weight (AjBW) rather than actual body weight. The basis for this recommendation has been that administered immunoglobulin does not distribute to body fat and is only present in the intravascular space and extracellular fluids. The use of adjusted body weight, rather than ideal body weight (IBW) or lean body weight (LBW) is based on the presumption that extracellular fluid is increased in patients with increased adipose tissue [8]. The descriptions and calculations of these derived body weight parameters are shown in the legend of Table 1.

**Table 1:** Showing actual body weight vs lean body mass, adjusted body weight and ideal body weight in kg

	Actual BW (kg)	BMI (kg/m^2^)	Lean BW (kg)	Adjusted BW (kg)	Ideal BW (kg)	SCIG/month (g)	Serum IgG (g/l)
Pre wt	132	43.1	65.4	92.4	66	57.6	7
Post wt	63	20.6				51.2	9.2
Pre SCIG/kg	0.44 (g)		0.88	0.62	0.87		
Post SCIG/kg	0.81 (g)						
**Pre serum IgG index (IgG/SCIG/kg)	15		7.95 (*predicted)	11.2 (*predicted)	8.05 (*predicted)		
**Post serum IgG index (IgG/SCIG/kg)	11.35		NA	NA	NA		

* The lean, adjusted and ideal body weights were computed from presurgical weight. The monthly SCIG dose is then expressed as a fraction of body weight as well as each of the calculated body weights. Finally, to account for the differences in steady state serum IgG an index ** was calculated serum IgG/(SCIG/kg) [7]. This is similar to the IgG efficiency index where the weight was static but the dose of SCIG/IVIG was adjusted in lean and obese patients [15]. In this case the dose of SCIG was static while the weight decreased. All measurements were trough levels, just prior to the next dose of SCIG.IVIG. The post bariatric serum IgG/(SCIG/kg) index of 11.35 correlated most closely with the adjusted body weight of 11.2. The IgG index was only calculated for predicted presurgical body weights (LBW, AjBW and IBW). Descriptions: The actual body weight (ABW) is the measured weight. The ideal body weight (IBW) is the most healthy weight for an individual taking into account gender, age and body build. The adjusted body weight (AjBW) in an obese person assumes 25% of fat tissue is metabolically active. In the case of SCIG/IVIG, it is assumed there is distribution to the increased extracellular fluid associated with adipose tissue. Lean body weight (LBW) is calculated when fat is subtracted from the total body weight. Formulae: IBW (women) = 45.5 + 2.3∗(height over 60 in.), AjBW = Ideal BW + (0.4∗(Actual BW − Ideal BW)), LBW 2005 (women) = 9.27∗103∗Actual BW8.78∗103 + (244∗BMI)

*LBW* lean body weight, *AjBW* adjusted body weight, *IBW* ideal body weight, *NA* not applicable

Here I report a patient with common variable immunodeficiency disorder (CVID) who underwent bariatric surgery for morbid obesity. Progressive weight loss was associated with gradual increases in her serum immunoglobulin levels allowing a reduction in dosage. As will be shown here, this observation supports the recommendation that the initial loading dose of IVIG, is most appropriately based on adjusted body weight and not actual body weight in PID patients.

## Case presentation

The 50 year old patient presented in 2011 with bronchiectasis and reduced immunoglobulin levels in 2011. Her IgG was 3.5 g/l (7–14), IgA < 0.07 g/l (0.7–4) and IgM 0.4 g/l (0.4–2.4). Vaccine responses were impaired to Pneumovax^®^, diphtheria, H. influenzae and tetanus toxoids (Table 2) [9]. She had reduced memory B cells and met the Ameratunga et al. criteria for CVID [10, 11] and qualified for intravenous immunoglobulin (IVIG). [12] One of her daughters has CVID but whole exome sequencing (WES) failed to identify the causative mutation in the family [13, 14]. Prior to IVIG, She was initially treated with prophylactic antibiotics but continued to have breakthrough infections. [12].

**Table 2:** Clinical features and laboratory investigations

Clinical symptoms	Recurrent infections Bronchiectasis
Family history of hypogammaglobulinemia	Daughter with CVID
Hb (130–190 g/l)	118
Neutrophils (1.9–7.5 × E + 9/l)	1.9
Platelets (150–400× E + 9/l)	208
IgG (7–14 g/l)	3.5
IgA (0.8–4 g/l)	< 0.07
IgM (0.4–2.5 g/l)	0.4
Autoantibodies	Nil
Immunophenotype	B cells present
Switched memory B cells (CD19+ CD27+ , IgD−: NR 5–21)	1.8
Tetanus pre/post (IU/ml)	0.64/0.52
Diphtheria pre/post (IU/ml)	0.03/0.08
HIB pre/post µg/ml	0.16/0.37
Pneumovax^®^ pre/post* (serotypes > 1.3 µg/ml/23)	0/0
Vaccine durability	Not done
Sequence variations	WES: no causative mutation
Relevant histology	Nil
T cell responses	Normal lectin responses Normal responses to tetanus, diphtheria and candida
Diagnosis	CVID (Ameratunga et al. criteria)
Treatment	IVIG/SCIG Bariatric surgery
Clinical outcome	Well

**Fig. 1:**
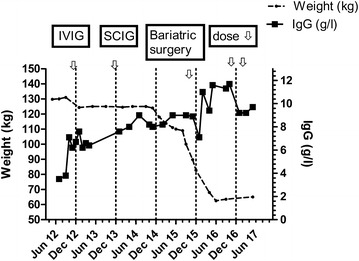
The patient’s peak weight was 132 kg. She was able to reduce her weight to 110 kg at the time of surgery in December 2015. She was initially treated with IVIG IN October 2012 and then changed to SCIG in October 2013. Her SCIG dose was 14.4 g every week. It was reduced to 13.6 g weekly in November 2016 and 12.8 g weekly in January 2017. Prior to weight loss, her trough immunoglobulin level was approximately 7 g/l. Her latest IgG in May 2017 was 9.2 g/l, in spite of a 20% reduction in SCIG dose. Arrows depict the changes in treatment

She had a peak weight of 132 kg and the initial immunoglobulin dose was calculated according to adjusted body weight. Thereafter, dosage was titrated according to her symptoms, which corresponded to a trough IgG of approximately 7 g/l. Following immunoglobulin replacement, there was a marked reduction in her sputum production and fewer exacerbations in her bronchiectasis. For lifestyle reasons, she was subsequently changed to SCIG treatment and has remained well (Fig. 1).

In spite of morbid obesity, she did not meet the stringent criteria for publically funded bariatric surgery in New Zealand, as she had not developed type 2 diabetes. Following discussion with the bariatric surgical team she was accepted for weight reduction surgery based on likely cost savings from reduced immunoglobulin requirements. She was able to lose 10 kg weight prior to bariatric surgery (Fig. 1). She underwent a roux-en-Y procedure and had an uncomplicated post-operative course.

Following bariatric surgery, she experienced progressive weight loss (Fig. 1) which has stabilised at 63 kg, which is slightly lower than her predicted ideal body weight (Table 1). Concomitant with weight loss, her trough IgG levels began to increase allowing a dosage reduction (Fig. 1). It is likely further reduction in SCIG dosage will be possible in the future. She remains in good health, with a marked improvement in sputum production and has been able to discontinue her prophylactic antibiotics.

## Discussion and conclusions

My observation offers direct evidence that SCIG requirements will decrease following substantial weight loss (Fig. 1). The patient’s actual weight decreased by 50% but her immunoglobulin requirements have decreased by approximately 20%. Her trough IgG levels were approximately 7 g/l prior to the surgery but increased to 11.7 g/l allowing a reduction in the SCIG dose. As can be seen from Table 1, her apparent SCIG dose/kg almost doubled from 0.44 g/kg/month to 0.81 g/kg/month with her weight loss. The reduced extracellular fluid following loss of adipose tissue is presumably the explanation for the reduced immunoglobulin requirements and increasing serum IgG. There may be other factors contributing to reduced SCIG requirements including amelioration of the inflammatory state associated with obesity and better control of her bronchiectasis [7].

I have used a calculated IgG index (serum IgG/SCIG dose/per kg ) to determine which derived body weight parameter prior to surgery correlated best with subsequent post weight loss SCIG requirements. This index accounts for changes in steady state serum IgG as well as changes in SCIG/IVIG dose/kg [7]. A version of this IgG index has been used to calculate changes in trough IgG levels when adjusting SCIG/IVIG doses in obese patients [15]. In my patient’s case the SCIG dose was initially kept steady, while her weight decreased.

Following weight loss her actual body weight IgG index (serum IgG/SCIG dose/per kg) was 11.35, which correlated closely with the predicted IgG index from the adjusted body weight (11.2), prior to weight loss (Table 1). The post weight reduction IgG index (11.35) takes into account the increased serum IgG and reduced SCIG dose following weight loss. As noted above, the decreased extracellular fluid compartment following loss of adipose tissue presumably explains the increasing serum IgG levels (Fig. 1). Other derived body weight parameters such as lean body mass and ideal body weight do not effectively model for this increased extracellular fluid compartment in obesity. They did not correlate closely with the post weight loss IgG index (Table 1).

The IgG index calculation in this patient supports basing initial loading doses of IVIG on adjusted body weight rather than actual body weight or ideal body weight (Table 1) in obese PID patients. As noted above subsequent maintenance SCIG/IVIG doses are based on the patient’s clinical response. A lower IVIG loading dose in obese PID patients has many advantages. There is an obvious fiscal benefit. The Hospital stay will shortened for the first visit and there are other advantages including a lower risk of thrombosis and cardiovascular adverse events from the large initial infusion [7].

Whether this observation indicates a lower IVIG dose can be used in obese patients with autoimmune or inflammatory disorders is less certain. As in other parts of the world, autoimmune and inflammatory disorders now account of the majority of IVIG usage in New Zealand [16]. Immunomodulatory IVIG doses are typically 2 g/kg, given over 2-5 days. The high peak IgG levels may be important for efficacy in autoimmune and inflammatory disorders compared with PIDs, where the steady state during maintenance may be more relevant. Recent studies from Canada provided data that using adjusted body weight can result in major cost savings [8, 17]. However, efficacy data was not provided, which is an important consideration, when adjusting IVIG doses. While there may have been immediate cost savings from a lower dose of IVIG, there is a risk of reduced efficacy, which could result in downstream costs to the health system [7]. Lower IVIG doses based on adjusted body weight in autoimmune and inflammatory disorders will have to be validated in future trials.

Obesity is currently a global public health problem. Bariatric surgery is being increasingly offered to patients with morbid obesity, when other interventions have failed. In these patients, there are many health benefits from weight reduction including reduced risk of diabetes, hypertension, coronary artery disease and osteoarthritis [18]. Generally patients with a BMI > 30 who have co-morbidities are considered suitable candidates for bariatric surgery. Co-morbidities qualifying for bariatric surgery include diabetes, hypertension and joint symptoms. In many countries, there are age limits also. Consensus criteria exclude patients with obesity secondary to endocrine disorders as well as those who have serious psychological disorders.

Bariatric surgical procedures are in evolution [18]. There are several surgical options including Roux-en-Y gastric bypass, sleeve gastrectomy, duodenal switch and adjustable gastric banding. Each of these procedures has its own advantages and disadvantages. Most procedures are carried out laparoscopically, which has many benefits, including reduced surgical morbidity. As seen here PID patients with bronchiectasis appear to tolerate this procedure well.

While there are many advantages of weight reduction, there are significant surgical risks and metabolic complications that must be factored when considering this option. Currently operative mortality is approximately three cases per 1000 [18]. Patients accepted for such surgical procedures undergo intensive counselling and are encouraged to undergo presurgical weight loss under the supervision of the dietician in the Bariatric surgical team. My patient was able to lose almost 10 kg prior to surgery (Fig. 1). Following surgery, she was seen regularly for nutritional advice and monitoring by the bariatric surgical team. Her weight has stabilised at approximately 63 kg and she no longer suffers from hypertension or pre-diabetes.

My observation shows that the absolute SCIG/IVIG requirements will substantially decrease following bariatric surgery in an individual patient. I have calculated a direct cost saving of $10,400 per year based on her reduced SCIG requirements. Over 30 years this saving alone will more than cover the cost of the procedure. She did not meet the strict NZ criteria for publically funded bariatric surgery but the economic argument presented here was an important factor in her qualifying for the procedure. The expected cost savings are materialising. The reduction in immunoglobulin requirements in future PID patients should be factored when a cost–benefit analysis is undertaken.

While this is a single observation, the patient served as her own historical control. This controls for patient related factors including bronchiectasis, genotype of the FcRn receptor etc. Given the increasing prevalence of obesity, there will be other PID patients receiving SCIG/IVIG who undergo weight reduction surgery in the future. It will be important to carefully document their weight loss and to compare it with their SCIG/IVIG requirements. Observations from such patients may be the most convincing evidence that adjusted body weight is the best parameter to base initial loading doses of IVIG in patients with PIDs.
